# Meaning of the spiritual aspects of health care in pregnancy and childbirth

**DOI:** 10.1590/1518-8345.5980.3774

**Published:** 2023-01-06

**Authors:** Dirce Stein Backes, Eronis Borges Gomes, Rosiane Filipin Rangel, Karla Maria Carneiro Rolim, Luciano Samaniego Arrusul, Josiane Lieberknecht Wathier Abaid

**Affiliations:** 1 Universidade Franciscana, Saúde/Enfermagem, Santa Maria, RS, Brazil.; 2 Scholarship holder at the Conselho Nacional de Desenvolvimento Científico e Tecnológico (CNPq), Brazil.; 3 Universidade Federal de Pelotas, Enfermagem, Pelotas, RS, Brazil.; 4 Universidade de Fortaleza, Enfermagem, Fortaleza, CE, Brazil.; 5 Universidade Franciscana, Saúde/Psicologia, Santa Maria, RS, Brazil.

**Keywords:** Nursing, Spirituality, Pregnancy, Parturition, Postpartum Period, Qualitative Research, Enfermagem, Espiritualidade, Gestação, Parto, Puerpério, Pesquisa Qualitativa, Enfermería, Espiritualidad, Embarazo, Parto, Periodo Posparto, Investigación Cualitativa

## Abstract

**Objective:**

to know the importance of the spiritual aspects of health care during pregnancy and childbirth, in the light of complexity thinking.

**Method:**

qualitative research, based on complexity thinking. Twenty-seven postpartum women with children between one month and six months old participated in the study. The data were collected between August and November 2021, based on individual interviews with guiding questions. Thematic analysis was used for data analysis.

**Results:**

three themes were obtained: The inseparability of spiritual care and emotional care; Connection between spirituality and the uterus - sacred temple; Alternative techniques for spiritual health care.

**Conclusion:**

the spiritual aspect of health care during pregnancy and childbirth can be considered an essential resource in the support of autonomy, security, and comfort. In addition, it can enable favorable outcomes in childbirth by strengthening the maternal-fetal attachment.

## Resumo

**Objetivo:**

conhecer o significado da dimensão espiritual do cuidado em saúde na gestação e no parto, à luz do pensamento da complexidade.

**Método:**

pesquisa qualitativa, fundamentada no pensamento da complexidade. Participaram 27 puérperas com filhos entre um mês e seis meses de idade. Os dados foram coletados, entre agosto e novembro de 2021, a partir de entrevistas individuais, com questões norteadoras. Utilizou-se para a análise dos dados a Análise Temática.

**Resultados:**

obtiveram-se três temáticas: A indissociabilidade entre cuidado espiritual e cuidado emocional; Conexão entre dimensão espiritual e útero - templo sagrado; Técnicas alternativas e complementares de cuidado espiritual em saúde.

**Conclusão:**

a dimensão espiritual do cuidado em saúde na gestação e no parto pode ser considerada recurso essencial no despertar da autonomia, da segurança e do conforto, além de possibilitar desfechos favoráveis no parto pelo fortalecimento do vínculo materno-fetal.

## Resumen

**Objetivo:**

conocer el significado de la dimensión espiritual de la atención en la salud durante el embarazo y el parto, a la luz del pensamiento de la complejidad.

**Método:**

investigación cualitativa, basada en el pensamiento de la complejidad. Participaron 27 puérperas con hijos entre un mes y seis meses. Los datos fueron recolectados entre agosto y noviembre de 2021, a partir de entrevistas individuales, con preguntas orientadoras. Para el análisis de los datos se utilizó el Análisis Temático.

**Resultados:**

se obtuvieron tres temas: La inseparabilidad entre el cuidado espiritual y el cuidado emocional; Conexión entre dimensión espiritual y útero - templo sagrado; Técnicas alternativas y complementarias del cuidado de la salud espiritual.

**Conclusión:**

la dimensión espiritual de la atención en la salud durante el embarazo y el parto puede ser considerada un recurso esencial en el despertar de la autonomía, la seguridad y el confort, además de posibilitar resultados favorables en el parto al fortalecer el vínculo materno-fetal.

**Highlights**

(1) Health care is conceived as a complex unit.

(2) Pregnancy and childbirth are a unique and complex path.

(3) Spiritual care for pregnant women contributes to favorable outcomes in childbirth.

(4) Spirituality gives meaning to human existence in any period of life.

(5) Nurses have the potential to expand their approach and encompass the multiple dimensions of care.

## Introduction

Pregnancy and childbirth are a unique and complex course^1^. Each pregnant woman is a different construct of physical, mental, social, and spiritual aspects. In the pregnancy course, the physical, mental, and social dimensions are easily recognized and considered in health care. The spiritual dimension, however, is superficially perceived, stimulated and considered by healthcare professionals^2^ ^-^ ^4^. Hence, the questions arise: what exactly is meant by health care? Is it possible to fragment the care of human beings - a complex system with bio-psycho-socio-spiritual centers?

A study shows that pregnancy and childbirth involve a mix of feelings such as pain, fear, anguish, uncertainties, joy and, at the same time, involve autonomous choices made by the pregnant woman. These choices are related to the preparation and type of delivery, companion of choice, basic care for the newborn, among others^5^. In parallel, another study shows that prenatal care needs to consider multi-dimensional and multi-professional care. From this perspective, the spiritual dimension represents a resource that enhances decisions and helps to overcome adverse events^6^.

The conception of Nursing as a science, art/technology of providing care for human beings/pregnant women in their uniqueness and multiple dimensions, in coordination with other professionals of health^7^ presumes that only an expanded and complex care is capable of prompting interactive and associative processes. Spirituality is a phenomenon that gives meaning to the different movements of human beings and to their own existence, through unique and indivisible experiences. Therefore, it is important that spirituality be welcomed and encouraged as an inseparable part of multi-dimensional care^8^ ^-^ ^9^.

Studies show that spirituality represents a prospective strategy for coping with existential pain and adversities. In cases of infertility, spirituality increases the ability of couples to overcome the existential void. In patients diagnosed with cancer, spirituality alleviates suffering and distress and nourishes hope. In patients on palliative care, spirituality creates and strengthens bonds between team members and patients/families, among other benefits^10^ ^-^ ^12^. In relation to pregnancy and childbirth, the question remains: what is the importance of the spiritual aspects of health care in pregnancy and childbirth?

In the search for a unique and multi-dimensional understanding of health care and wishing to contribute to the institutionalization of approaches that perceive, integrate and expand, the objective of this study is to know the importance of the spiritual aspects of healthcare during pregnancy and childbirth, in the light of complexity thinking.

In this study, the term *complexity* is understood as everything that is woven together and that evokes at least more than one circumstance or possibility of interaction. It is understood that both the knowledge of the whole depends on the parts, and the knowledge of the parts depends on the knowledge of the whole^13^ ^-^ ^14^.

## Method

### Type of study

Qualitative research based on complexity thinking and guided by the uniqueness and multiple dimensions of health care. We aim to develop a methodological path in which the researcher is induced to learn, invent, and recreate their own path, through interpretative and significant processes in the here and now^15^.

### Setting, participants, and selection criteria

The study was carried out with 27 postpartum women from a city in the central region of the state of Rio Grande do Sul, Brazil. This city has 34 health centers, of which 20 are family health strategy centers and 14 are traditional primary care center.

Postpartum women with children from one to six months of age and who had participated in groups of pregnant women in one of the 14 primary care centers were included in the study. This period was selected because it is understood that, in the family context, the first month after the arrival of the baby brings significant changes. Postpartum women under 18 years of age, with children older than six months, or unavailable for data collection on the previously scheduled time were excluded.

### Data collection technique and period 

Data were collected between August and November 2021. After applying the inclusion and exclusion criteria, 27 postpartum women participated in the research. The participants were indicated by the professionals of the primary care centers, based on prenatal consultations in these centers. With the names and addresses of those interested, the researchers sent a formal invitation, with a schedule of days and times for data collection. Data were obtained by a trained professional, author of this study, through individual interviews with guiding questions that were addressed in depth.

The interviews were carried out at the place, day and time indicated by the participants. Whenever possible, the interviews occurred in a welcoming and quiet environment. The questions that guided the interviews were: What would you highlight the most during pregnancy and childbirth? What do you understand by health care during pregnancy and childbirth? Do you think it is important to address the spiritual aspect of health care during pregnancy and childbirth? If yes, how can this be done? It should be noted that the professional interviewer had previous experience in leading groups of pregnant women, which favored the dialogue with the participants.

The interviews had an average duration of 30 minutes and were recorded on a digital recorder for subsequent analysis. The process considered the uniqueness of each participant, so that they could express their perceptions with security and tranquility. After being organized, the interviews were fully transcribed by two researchers using a text editor. The transcribed data set resulted in 79 pages.

### Data processing and analysis

Thematic Analysis was used as an analysis technique, systematized in six stages: Data familiarization - the researcher was immersed in the data to become familiar with the content in depth and breadth; Inductive generation of initial codes - at this stage, the researcher manually and systematically coded the data set, with full and equal attention to each item; Generation of themes - at this stage, the different codes were clarified according to the proposed theoretical framework, in order to combine them into comprehensive themes; Reviewing the themes - at this stage, the researcher refined the themes, based on a pattern that showed things in common and clear distinctions between the themes; Definition of the themes - The themes were (re)defined, identifying the essence of each of them and of the set of themes; Writing of the research report - it consisted of the final analysis and writing of the report^16^ ^-^ ^17^. This analysis process, however, was not isolated and linear; it required a recursive approach, going back-and-forth between stages, as necessary.

Data analysis started with patterns of meanings, which occurred during data collection, that is, during the conduction of the interviews. Throughout the process, the constant recording of ideas, insights, drafts, and schemes was valued, not for accuracy, but with the purpose of enabling a deep immersion in the data.

### Ethical aspects in research

To comply with ethical guidelines, the recommendations of Resolution No. 466/2012 of the National Health Council were followed. To maintain anonymity, the speeches of the participants were identified throughout the text by the letter “P” (Participant), followed by an Arabic number according to the order of the speeches: P1, P2... P27. The research participants were informed about the objectives, the methodology used, the right of free access to the data, and the possibility of withdrawing at any time. Their consent and acceptance were given through the signing of the Informed Consent Form. The project was approved by the research ethics committee under number: 4,253,922; CAAE: 53319116.5.0000.5306. 

## Results

The mean age of the 27 postpartum women was 31 years old, the mean number of pregnancies was 3, and the mean level of education was complete high school. Among the participants, eight had had a normal delivery and 19 had had a c-section. Only seven had a companion of choice at birth. The four most prominent professions among the participants, in descending order, were: nursing technician, student, housewife and teacher.

The organized and analyzed data resulted in three themes: The inseparability of spiritual care and emotional care; Connection between spirituality and the uterus - sacred temple; Alternative techniques for spiritual health care.

### The inseparability of spiritual care from emotional care

The speeches of the participants revealed the fine line between the meanings attributed to spiritual and emotional care, sometimes confusing and, at other times, very similar, as they report that spiritual health care provided comfort, encouragement, and emotional support, bringing peace and serenity for pregnancy and childbirth: *Spirituality gives emotional support, brings inner peace and alleviates the anxiety, worry and stress from this transition period that is being a first-time mother.* (P3)

Pregnancy is a unique moment in a woman’s life, and in this context, spirituality is understood as a phenomenon that gives meaning to life: *I believe that spirituality is important during pregnancy and childbirth. The birth process is a unique moment in a woman’s life, in which many feelings and emotions are present.* (P7)*. Spirituality uplifts our thoughts so that we can maintain better emotional balance, have a healthier pregnancy and a safe and peaceful delivery.* (P13)*.*

An even greater importance was attributed to spirituality by the participants who referred to the pregnancies as unwanted and/or with complications. For these women, spirituality represents spiritual and mental support: *Women go through very intense physical, emotional, and psychological changes during pregnancy, and it is very difficult if they do not have spiritual and mental support. It is quite delicate, especially if the woman/couple goes through an unplanned pregnancy or the woman has several complications during pregnancy.* (P17)*. Sometimes, pregnancy is just a process that a woman must go through, but that not necessarily means that she will be a mother at the end of this process. Therefore, it would be interesting to offer, in addition to some specialties, spiritual and psychological support.* (P12)

Some participants mentioned anguish, uncertainties and fears during the birth process and described how emotionally shaken they felt. In this context, they reported that spiritual health care should be considered an important resource to deal with suffering and, therefore, it should be available to all users, especially future parents: *Everyone should have access to spiritual care and to the cultivation of spirituality, especially couples that choose to have children, who should have access to knowledge even before pregnancy, so that they can cope with emotional issues and bear children. I search for this in therapies and books, but not everyone has this kind of access.* (P11)

Although most participants highlighted the importance of the spiritual aspect of health care during pregnancy and childbirth, this aspect is still little investigated in the social environment. The participants reported that the connection with spirituality must be seen as sacred, as the woman’s body is a sacred temple, capable of prompting spiritual connection with the being it is generating: *Before this pregnancy I was depressed. It was very difficult to understand what was happening. In the second pregnancy, by working on spirituality, motherhood became happier, as I perceived the blessing of receiving such a perfect little being, which only brings happiness. In the case of pregnant women, who are more sensitive, it is advisable to care and to bring comfort and tranquility. After all, for most women, this is the happiest time of their life.* (P9)

At the same time that they recognized the relevance of the spiritual aspect of care, the participants emphasized that spirituality cannot be imposed, as it is part of the human experience, especially in unique moments such as pregnancy and childbirth. From this perspective, the participants noted that spirituality is not reduced to linear and isolated moments but is a circular movement that can coordinate different vital aspects: *I think that the spiritual aspect, by its nature, cannot be imposed, especially at a critical moment like this, when something very important happens, and when we feel vulnerable and exposed (with the medical team, the devices and procedures). I think that, as a part of life, the spiritual aspect will be present at the time of delivery, in an individual way, according to what each one believes. During childbirth, in difficult moments of pain, there was a person (from the nursing team) who held my hand and massaged my back trying to soothe me. That presence was so caring, friendly, and empathic that it was fundamental, and left precious marks.* (P21)

The participants reported that the spiritual aspect of healthcare can and should be studied and considered by all professionals in the area, even if it is confused with emotional care. Professionals must have experience in spiritual health care, making room for empathy and recognizing unique beliefs, rituals and symbols related to pregnancy and childbirth.

### Connection between spirituality and the uterus - sacred temple

The report of most of the participants showed that the search for the spiritual aspect of health care is associated with the ability to believe in a higher power, out of a connection that does not happens in everyday life, but is felt deeply (the baby in the womb). The participants acknowledged that the new being allows deep connections that transcend the reality that can be perceived or explained. Under this approach, the spiritual aspect of health care is reinforced as a resource that enhances the maternal-fetal attachment: *Spirituality is everything in the life of a pregnant woman. Fear, insecurity, and uncertainties make us seek spirituality looking for reassurance and connection with the new being. With this strong connection, we feel safer and more encouraged to endure the pain and to promote the well-being of the baby. Every woman has her own way of connecting and finding spirituality.* (P5)*. It’s a perfect connection. The baby feels everything the mother feels. During pregnancy and at the time of delivery, I always asked for protection for my son, and I always felt relieved.* (P14)

Birth is considered a sacred and magical phenomenon, which cannot be explained through linear and one-dimensional perceptions. The participants recognize that life, pregnancy and childbirth can only find meaning in spirituality: *I think is extremely important to be connected with spirituality, especially in this sacred moment that is to generate a new life* (P13)*. The mother and baby are in a transcendent connection. Birth must be seen as sacred.* (P20)*. This moment is magical and unique in a woman’s life. She must seek to be confident and at peace from the very start of pregnancy.* (P27)

In the participants’ perception, pregnancy and childbirth allow a deep connection between the spiritual realm and the uterus - a sacred temple where the new being lives. It is important, therefore, to stimulate and encourage the pregnant woman to see herself in this cosmic and transcendent manner. *When we are pregnant, our body becomes a sacred temple for another being. If you are in spiritual communion, all this positive energy will go through and lead the baby to a happy birth. Those on a higher spiritual level tend to have a better pregnancy and delivery.* (P18)

Although spirituality represents a special aspect in the lives of pregnant women, this process is still superficially addressed by nurses and other health professionals. The participants reported that prenatal consultations were merely informative and prescriptive, and they were rarely heard and welcomed in their beliefs, values and expectations: *Fear and insecurity are feelings that stick to us during pregnancy, so we seek spirituality. But in prenatal care, rarely does anyone ask and talk about it. Every woman has to find her own way of connecting and finding spirituality.* (P1)*. I had depression; it was difficult to understand what was happening. It would be good to receive guidance on prenatal care not to focus on bad thoughts and feelings of anger and sadness.* (P5)

The participants’ reports showed the desire for the spiritual aspect of health care to be considered and addressed during pregnancy and childbirth, to provide relief from suffering and to strengthen the connection between mother and baby. In the understanding of the participants, the spiritual aspect of health care is not reduced to isolated and simplified approaches; it requires welcoming attitudes that can promote and strengthen transcendent connections.

### Alternative techniques for spiritual health care

When asked if the spiritual aspect of health care during pregnancy and childbirth was important, all participants gave affirmative answers. The participants highlighted the benefits of alternative and complementary health care techniques with emphasis on spirituality, which provides feelings of communion and enlightenment. Among the alternative techniques, the use of reiki, incense, music, meditation and dimmed lights during childbirth were highlighted. *Reiki and other alternative techniques could be used. This way, many mothers would not have postpartum depression. I had it and if they did this spiritual work and other alternative therapies, I wouldn’t have had it. This whole process of being a mother messes with our feelings, with our soul.* (P14)*. Each person has their beliefs and their level of connection to the universe. At the time of delivery, it would be good to dim the lights, light an incense.* (P18)*. The feelings of suffering, fear and loneliness can be relieved through listening and comforting, which consequently enhance spiritual care and minimize postpartum disorders.* (P21)

Several participants mentioned the importance of conversation circles and therapeutic groups held in primary care centers, which sometimes include spiritual health care. Under this approach, they reinforced the relevance of prenatal care, an opportunity in which pregnant women can develop a closer and more effective bond with health professionals: *Therapies and conversation circles with trained professionals are very important.* (P19)*. The doctors could do it, which is rare, or the nurses, in prenatal consultations. Another possibility would be to create conversation groups in health centers and even in hospitals, focusing on topics such as spirituality and others.* (P2)*. There is a greater connection between the baby and spirituality when the baby is still in the womb. So, these moments could be better spent in prayers, meditations, and conversation circles. These are means that strengthen and maintain the balance of body, mind, and spirit.* (P6)

At the same time, the participants mentioned alternative healthcare techniques, such as reiki, music and dance, practices detached from specific religions, which are not yet employed at healthcare services: *I think techniques such as music, dance, and reiki are important. There isn’t anything like this available for pregnant women, not that I’m aware. What resembles this is too tied to specific religions. Being pregnant or in a vulnerable situation does not mean that you will want a religion. So, I think it’s something that can be improved on prenatal care.* (P23)

The alternative health techniques desired by the participants cannot, however, be imposed by the professionals. It is important that each pregnant woman is heard and welcomed in her singularity and according to her needs: *Alternative therapies are very important, but they must be applied according to the beliefs and expectations of each woman and regardless of their religion. It is necessary to see the individuality and wishes of each woman.* (P26)

There is an expressed and/or unexpressed desire for alternative therapies in the participants’ speech. These therapies are seen as resources that can balance mind/body/spirit and that should be widely adopted in health services. It is essential, therefore, to overcome interventionist and one-dimensional approaches, to provide health care centered on the user - the subject of the action.

## Discussion

Important progress in the quality of maternal and child health care has been achieved in the last two decades. However, maternal and neonatal mortality rates decrease slowly. Studies^18^ ^-^ ^21^ show that pregnancy is a period that requires attention, as it can affect the pregnancy-childbirth course both positively and negatively. In this context, new possibilities and assets that consider both the uniqueness and the multiple dimensions of health care during pregnancy and childbirth are needed.

In this context, the spiritual aspect of health care has been gaining importance in pregnant women’s care, as it can have a beneficial impact on the outcome of childbirth. Spiritual health and attitudes related to maternal-fetal attachment are considered promising strategies in the process of adaptation and healthy development of pregnancy. In this sense, a study^22^ suggests increasing the spiritual aspect of health care, as a means to strengthening maternal-fetal attachment.

However, the spiritual component cannot be confused with religiosity. Although they are widely used as synonyms, religion and spirituality have distinct and complementary meanings. While the former has a formal and organized character, the latter is characterized as a phenomenon inherent to human experience, with a diversity of meanings, which may or may not be of a religious nature^23^. Spirituality is related to the ability to overcome one’s own limitations and to give new meanings to human existence and life experiences in different periods^24^.

Even though they could not clearly define spirituality or spiritual health care, the participants in this study showed connections and meanings that are not reduced to the biological, mental, and social dimensions. This perception was more evident when they mentioned the connection between spirituality and the meaning attributed to human existence, which is not restricted to material and random thoughts. This idea is confirmed by scholars who show that mystical experiences induce positive feelings and emotions, capable of releasing underlying energies and stimulating connections with the sacred and the transcendent^25^ ^-^ ^26^.

Another relevant aspect that emerged in this study refers to the relationship between the spiritual connection and the maternal uterus/sacred temple where the new being lives. Despite the lack of evidence on this association, the results indicate that nursing/health professionals should increase their awareness, in order to understand healthcare in its multiple aspects, that is, beyond the physical aspect. In this context, the Nurse has the decisive and proactive role of identifying unique care needs, as well as promoting and protecting the health of pregnant women, in its different dimensions and expressions of reality^7^.

According to scientific evidence, the pregnancy-postpartum period requires professional approaches that go beyond interventionist and biomedical models^24^ ^-^ ^25^. In the process of generating a new being, the pregnant woman experiences fear, uncertainty, and anguish combined with a mix of expectations, dreams, and achievements. In this context, it is essential that the pregnant woman is welcomed, supported, and strengthened in her initiatives and life perspectives throughout the whole pregnancy process. Nursing/healthcare professionals, especially those who work in prenatal care, must be aware of the different movements and feelings that are encompassed in pregnant women’s process, so that they can encourage them to be the protagonists of their own story^27^.

A study recognizes the spiritual aspect as inherent to the human being, as it is characterized by unique experiences that enable connections with oneself, with others, and with the transcendent^24^. In this context, the spiritual aspect of health care can enable circular and interactive possibilities in pregnancy and childbirth, enhance convictions, and minimize adverse events.

Therefore, pregnancy and childbirth are phenomena that allow pregnant women to experience spirituality in a more or less intense way, depending on their characteristics, trajectories, and personal experiences. These experiences are influenced by their socioeconomic, cultural, and religious background, which can influence the outcome of childbirth.

Health care can only be understood in the light of complexity thinking, which requires acceptance and respect for the multiple aspects it can have. The construction of multi-dimensional/multi-professional care requires knowledge and practices that go beyond the mechanical work and that consider individual values, beliefs, and convictions^12^.

In the perspective of complexity, the conception of a unique and multi-dimensional care for pregnant women goes back to a historical and hegemonic tradition in the obstetric area, characterized by prescriptive and hierarchic practices, in which a professional relationship of subject-object prevailed. In this traditional model, the pregnant woman did not have the autonomy to make her own decisions. However, spiritual health care is always unique and multi-dimensional, that is, it is complex and made from everything that is woven together and that evokes more than one circumstance or interactive and associative possibility^10^ ^-^ ^11^.

The contributions of this study to the advancement of Nursing science are related to the awareness that nursing professionals have the potential to expand their perception and to develop spiritual health care, along with the other members of the multi-professional health team. The present study may also be seen as a stimulus for other researchers who intend to address the multi-dimensional and multi-professional nature of health care.

A limitation of this study is the insecurity of some study participants during the individual interviews. Although the COVID-19 contingency plan was followed, a feeling of insecurity due to pandemic was perceived in some interviews, but this did not hinder the research.

## Conclusion

The spiritual aspect of health care during pregnancy and childbirth can be considered an essential resource in the support of autonomy, security, and comfort. In addition, it can enable favorable outcomes in childbirth by strengthening the maternal-fetal attachment. Therefore, spirituality is a phenomenon that gives meaning to each stage of life, while also giving meaning to human existence itself.

It is imperative to overcome the logic of fragmented, linear, prescriptive, and one-dimensional health care. The understanding of care as a complex unit - unique and multi-dimensional - is essential in the process of encouraging autonomy, creativity, interactivity and close, dialogical, and humanized relationships.

The present research emphasizes the importance and recommends further studies on the spiritual aspect of health care during pregnancy and childbirth, so that spirituality may become a knowledge that can be employed in the professional’s daily practice, especially in the context of primary health care.
